# Glucose oxidase-like activity of cerium oxide nanoparticles: use for personal glucose meter-based label-free target DNA detection

**DOI:** 10.7150/thno.41484

**Published:** 2020-03-15

**Authors:** Hyo Yong Kim, Ki Soo Park, Hyun Gyu Park

**Affiliations:** 1 Department of Chemical and Biomolecular Engineering (BK21+ Program), KAIST, 291 Daehak-ro, Yuseong-gu, Daejeon 34141, Republic of Korea.; 2 Department of Biological Engineering, College of Engineering, Konkuk University, Seoul 05029, Republic of Korea.

**Keywords:** Polymerase chain reaction, Cerium oxide nanoparticle, Glucose oxidase-like activity, Personal glucose meter, Biosensor

## Abstract

Recently, personal glucose meter (PGM) has been utilized for the detection of non-glucose targets for point-of-care (POC) testing. Aimed at this goal, we herein developed a new PGM-based label-free read-out method for polymerase chain reaction (PCR) based on our novel finding that cerium oxide nanoparticles (CeO_2_ NPs) exhibit glucose oxidase-like activity comparable to the natural glucose oxidase enzyme.

**Methods**: In principle, DNA amplicons produced by PCR in the presence of target DNA electrostatically bind to CeO_2_ NPs, leading to their aggregation and reducing the efficiency for CeO_2_ NP-catalyzed glucose oxidation reaction. Thus, glucose is hardly oxidized to gluconic acid, resulting in the maintenance of initial high glucose level. On the contrary, in the absence of target DNA or presence of non-target DNA, DNA amplicons are not produced and glucose is effectively oxidized by the glucose oxidase-like activity of CeO_2_ NPs, leading to the significant reduction of glucose level. Finally, the resulting glucose level is simply measured by using PGM.

**Results**: With this strategy, DNA amplicons were quantitatively examined within *5* min, realizing ultrafast analysis of PCR results without any cumbersome and labor-intensive procedures. In addition, the target genomic DNA derived from *Escherichia coli* (*E. coli*) was sensitively determined down to *10* copies with high selectivity.

**Conclusion**: Importantly, the use of PGM as a detection component enables its direct application in POC settings. Based on the meritorious features of PGM such as rapidity, simplicity, and cost-effectiveness, we expect that the devised system could serve as a core platform for the on-site read-out of PCR amplification.

## Introduction

Personal glucose meter (PGM) that enables the self-monitoring of blood glucose level has been widely utilized as the home-kit for the self-diagnosis of diabetes. In recent years, many researchers have attempted to use PGM for the detection of non-glucose targets at point-of-care (POC) setting based on its advantageous features such as cost-effectiveness, simplicity, and portability 1-14. The pioneering work was executed by Xiang et al. for the detection of various non-glucose analytes, which relies on the correlation between the concentration of non-glucose target and glucose level 15. Specifically, the invertase-conjugated DNA probe was hybridized with DNA aptamer. Only in the presence of non-glucose target that forms the complex with DNA aptamer, the invertase-modified DNA probe was released and then used to catalyze the hydrolysis of sucrose to glucose after magnetic separation. The resulting glucose level that is proportional to the amount of non-glucose target was finally measured by using PGM. Based on this principle, diverse non-glucose analytes such as cocaine, adenosine, interferon-gamma, and uranium have been determined. However, this strategy still has the critical limitations that it requires the immobilization of DNA aptamer on the magnetic beads, the conjugation of DNA probe with invertase, and magnetic separation steps, which make the total assay quite complex and limit the practical application of PGM for biomolecular detection in facility-limited environments.

Since the discovery that magnetic nanoparticles (MNPs) have the peroxidase-mimicking activity, various metal nanoparticles including gold nanoparticles (AuNPs), platinum nanoparticles (PtNPs), cupric oxide nanoparticles (CuO NPs), iron oxide nanoparticles (Fe_3_O_4_ NPs), and cerium oxide nanoparticles (CeO_2_ NPs) have been found to possess the enzyme-mimicking activities such as peroxidase, catalase, and oxidase-like activities, which are even superior to those of natural enzymes 16-29. Importantly, CeO_2_ NPs with oxidase-like activity exhibit the high stability and catalytic efficiency for substrate oxidation even without additional oxidizing agent. In addition, it was reported that the enzyme-mimicking activity of metal nanoparticles can be regulated by nucleic acids that bind to metal nanoparticles by the electrostatic interaction, inducing the aggregation of metal nanoparticles and thus decreasing their catalytic activity 24,26.

In this work, we developed a new PGM-utilized method for the on-site read-out of polymerase chain reaction (PCR) result based on our novel finding that CeO_2_ NPs exhibit the glucose oxidase-like activity enabling the correlation of DNA amplicon concentration with glucose level. In principle, the DNA amplicons produced by PCR in the presence of target DNA bind to CeO_2_ NPs through electrostatic interaction, inducing their aggregation and, accordingly, reducing the efficiency for CeO_2_ NP-catalyzed glucose oxidation reaction. Thus, the effective conversion of glucose to gluconic acid by CeO_2_ NPs is suppressed, keeping the initial high glucose level that can be simply measured by PGM. With this strategy, the target DNA was sensitively determined down to *10* copies with high selectivity. More importantly, the detection of DNA amplicons was completed in less than *5* min based on the high catalytic activity of CeO_2_ NPs, accomplishing ultrafast analysis of PCR results without any laborious and complex step by simply utilizing PGM as a detection component.

## Methods

### Materials

All DNA oligonucleotides used in this study were synthesized and purified with high performance liquid chromatography (HPLC) by Bioneer^®^ (Daejeon, Korea). The sequence information of oligonucleotides employed in this study is in Table S1. The personal glucose meter (PGM) whose dynamic range for glucose detection is from 0.6 to 33 mM was purchased from Accu-Chek (Roche, Basel, Switzerland). Cerium (IV) oxide nanoparticle (CeO_2_ NP), sodium acetate, glucose, ethidium bromide (EtBr), hydroethidine, and 2,5-dihydroxybenzoic acid (DHB) were purchased from Sigma-Aldrich (St. Louis, MO, USA). The deoxynucleoside triphosphate (dNTP) and i-Taq^TM^ DNA polymerase were purchased from Intron Biotechnology Inc. (Daejeon, Korea). Ultrapure DNase/RNase-free distilled water (DW) purchased from Bioneer^®^ was used in all experiments. All chemicals used in this study were of analytical grade.

### PGM-based label-free method for the read-out of PCR amplification using glucose oxidase-like activity of CeO_2_ NP

PCR for the amplification of 16S rRNA gene in *Escherichia coli* (*E. coli*) genomic DNA (gDNA) was first carried out on S1000^TM^ thermal cycler (Bio-Rad, CA, USA) in a PCR solution (25 μL) containing 0.2 mM each dNTP, 0.4 μM each primer, 2.5 U i-Taq^TM^ DNA polymerase, *E. coli* gDNA at different copy numbers, and 1X PCR buffer (10 mM Tris-HCl (pH 8.3), 50 mM KCl, and 2 mM MgCl_2_). The PCR solution was heated up to 95 °C for 5 min followed by 40 cycles of 95 °C for 15 s, 58 °C for 15 s, and 72 °C for 30 s and finalized at 72 °C for 5 min. The DNA amplicon (524 bp) was purified using NucleoSpin purification kit (Macherey-Nagel, Duren, Germany) according to the manufacturer's protocol. Next, the purified DNA amplicon (25 μL) was added to CeO_2_ NP solution containing 2.5 μL CeO_2_ NP (2 wt%) and 12.5 μL sodium acetate buffer (0.4 M, pH 4.2) and the mixture was incubated at room temperature for *2* min. After the addition of *10* μL glucose (*300* mM), the reaction solution was further incubated at room temperature for *1.5* min. Finally, the resulting glucose level was measured by PGM. For the determination of *E. coli* gDNA in the human serum sample, PCR solution (*25* μL) was first prepared by adding *0.2* mM each dNTP, *0.4* μM each primer, *2.5* U i-Taq^TM^ DNA polymerase, *E. coli* gDNA at different copy numbers, and 1X PCR buffer (10 mM Tris-HCl (pH 8.3), 50 mM KCl, and 2 mM MgCl_2_) into 1% human serum sample, which was then analyzed according to the same procedure described above.

### Investigation of glucose oxidase-like activity of CeO_2_ NP

The solution prepared by mixing 30 μL DW, 12.5 μL sodium acetate buffer (0.4 M, pH 4.2), 2.5 μL CeO_2_ NP (2 wt%), and 5 μL hydroethidine (10 μg/mL) was incubated at room temperature for 1.5 min. The fluorescent signal from hydroethidine was scanned in the range from 570 to 670 nm with 530 nm excitation wavelength by utilizing Tecan Infinite M200 pro-microplate reader (Mnnedorf, Switzerland). In addition, the solution for the matrix-assisted laser desorption/ionization time-of-flight (MALDI TOF) analysis prepared by mixing 25 μL DW, 12.5 μL sodium acetate buffer (0.4 M, pH 4.2), 2.5 μL CeO_2_ NP (2 wt%), and 10 μL glucose (300 mM) was incubated at room temperature for 1.5 min. Then, the solution was mixed with equal volume of matrix prepared by resolving 10 mg/mL DHB into 50% ethanol and the mixture was loaded on MTP 384 ground steel MALDI TOF MS plate (Hudson Surface Technology, NJ, USA). After drying of the loaded sample at room temperature for overnight, MALDI TOF analysis was conducted utilizing Autoflex III MALDI-TOF MS instrument equipped with ND-YAG laser operated at 355 nm wavelength (Bruker Daltonics, Germany). The voltage, delayed extraction time, grid voltage, laser rate, and laser shots were 20 kV, 10 ns, 70%, 1 - 200 Hz, and 1,500 per sample spot, respectively.

The kinetic parameters of CeO_2_ NP-catalyzed glucose oxidation reaction were obtained from the Michaelis-Menten model. The reaction velocity defined as L_0_ - L where L_0_ and L are glucose levels measured by PGM before and after CeO_2_ NP-catalyzed glucose oxidation reaction (30 min), respectively, was calculated from the solutions prepared by mixing 17.5 μL DW, 12.5 μL sodium acetate buffer (0.4 M, pH 4.2), 5 μL CeO_2_ NP (700 nM), 10 μL purified DNA amplicon (500 nM), and 5 μL glucose at varying concentrations and then incubated at room temperature for 30 min.

### Confirmation of DNA-induced aggregation of CeO_2_ NP

We examined the crystalline nature of the employed CeO_2_ NP by using SmartLab X-ray diffractometer (Rigaku, Tokyo, Japan) with monochromatic Cu-Kα radiation. The scan range and rate were 20° - 65° and 5°/min, respectively. The surface state of CeO_2_ NP was also analyzed by using X-ray photoelectron spectrometer (Sigma Probe, Thermo Fisher Scientific, MA, USA) with microfocused monochromator X-ray source. In addition, the transmission electron microscopy (TEM) and energy-dispersive X-ray spectroscopy (EDS) data were obtained by using Cs-corrected STEM (JEM-ARM200F) in National NanoFab Center (NNFC, Daejeon, Korea). Samples for TEM and EDS analyses were prepared by casting 15 μL sample onto the copper grid (300 mesh) with a lacey carbon film (LC300-CU) purchased from Electron Microscopy Sciences (Hatfield, PA, USA). Then, the loaded sample was dried at room temperature overnight before the analysis. In addition, the zeta potential and dynamic light scattering (DLS) analysis were performed by using Zetasizer (Malvern, PA, USA). For zeta potential and DLS measurement, *1* mL sample prepared by adding 0.5 mg/mL CeO_2_ NP and 100 nM purified DNA amplicon into 0.1 M sodium acetate buffer (pH 4.2) was added in Zetasizer capillary cell and polystyrene cuvette, respectively, and the samples were scanned three times to obtain average zeta potential and diameter of CeO_2_ NP. The solution for the ultraviolet-visible spectroscopy (UV-Vis) analysis was prepared by mixing 25 μL DW, 12.5 μL sodium acetate buffer (0.4 M, pH 4.2), 2.5 μL CeO_2_ NP (2 wt%), and 10 μL purified DNA amplicon (500 nM). After the incubation at room temperature for 2 min, the solution was centrifuged at 10,000 g for 5 min. The precipitated CeO_2_ NP/DNA complex was dispersed in 0.1 M sodium acetate buffer (pH 4.2) and then the absorbance spectrum in the range from 220 to 440 nm was measured utilizing NanoDrop^TM^ One (Thermo Fisher Scientific, MA, USA).

## Results & Discussion

### PGM-based label-free method for the read-out of PCR amplification using glucose oxidase-like activity of CeO_2_ NP

**Figure 1:**
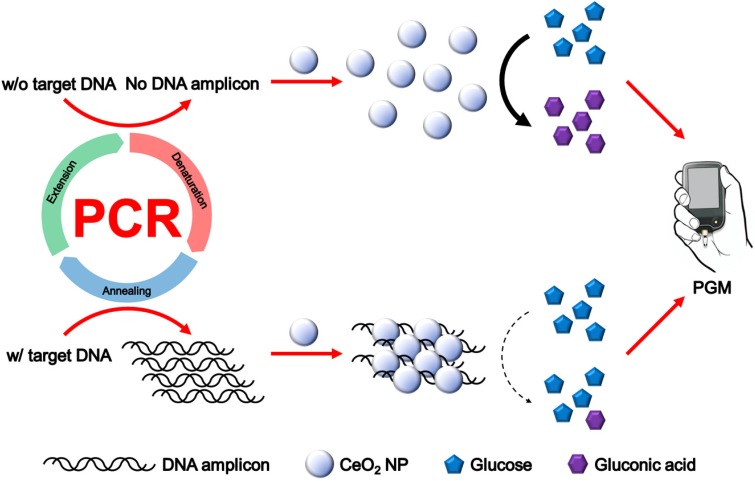
Schematic illustration of PGM-based label-free read-out of PCR amplification using glucose oxidase-like activity of CeO_2_ NP.

The overall procedure for PGM-based label-free read-out of PCR amplification using glucose oxidase-like activity of CeO_2_ NPs is illustrated in Figure 1. The reaction system involves the PCR amplification with the target specific primer sets followed by the incubation with CeO_2_ NPs. In the presence of target genomic DNA (gDNA) derived from *Escherichia coli* (*E. coli*), the PCR process generates DNA amplicons that bind to CeO_2_ NPs through the electrostatic interaction with positively charged surface of CeO_2_ NPs, which induces the aggregation of CeO_2_ NPs. As a result, the glucose substrate has a limited access onto CeO_2_ NPs and initial high glucose level is maintained. In contrast, in the absence of target gDNA, DNA amplicons are not produced by PCR process. Thus, the CeO_2_ NPs are well dispersed in the solution and thus exert the effective glucose oxidase-like activity. Accordingly, glucose is effectively converted to gluconic acid, leading to significant reduction of glucose level. Finally, the resulting glucose level that is proportional to the amount of produced DNA amplicon is simply measured by using PGM.

### Investigation of glucose oxidase-like activity of CeO_2_ NP

Since this is the first attempt to discover and utilize the glucose oxidase-like activity of CeO_2_ NPs, we examined the mechanism of CeO_2_ NP-catalyzed glucose oxidation reaction. As described in the following equation, the auto-catalytic redox switching of two oxidation states, Ce^3+^ and Ce^4+^, on the surface of CeO_2_ NP would be the key for the glucose oxidase-like activity of CeO_2_ NP 30-33.

2 CeO_2_ (Ce^4+^) + 2 H^+^ (aq) → Ce_2_O_3_ (Ce^3+^) + H_2_O (1)

Ce_2_O_3_ (Ce^3+^) + 1.5 O_2_ → 2 CeO_2_ (Ce^4+^) + O_2_•^-^ (2)

O_2_•^-^ + C_6_H_12_O_6_ + 2 H^+^ (aq) → C_6_H_12_O_7_ + H_2_O (3)

O_2_•^-^ + Ce_2_O_3_ (Ce^3+^) + 2 H^+^ (aq) → 2 CeO_2_ (Ce^4+^) + H_2_O (4)

Under the acidic condition, the surface of CeO_2_ NP (Ce^4+^) is first reduced to Ce_2_O_3_ (Ce^3+^), generating oxygen vacancy (equation 1). Then, the oxygen molecule (O_2_) adjacent to the surface adsorbs onto the oxygen vacancy in Ce^3+^ state while being reduced to superoxide anion radical (O_2_•^-^) with the concomitant oxidation of Ce^3+^ to Ce^4+^ (equation 2). Next, O_2_•^-^, a powerful oxidizing agent, catalyzes the oxidation of glucose to gluconic acid (equation 3) and Ce^3+^ to Ce^4+^ (equation 4). We assume that this auto-catalytic redox reaction of CeO_2_ NP induces highly efficient glucose oxidation process.

**Figure 2:**
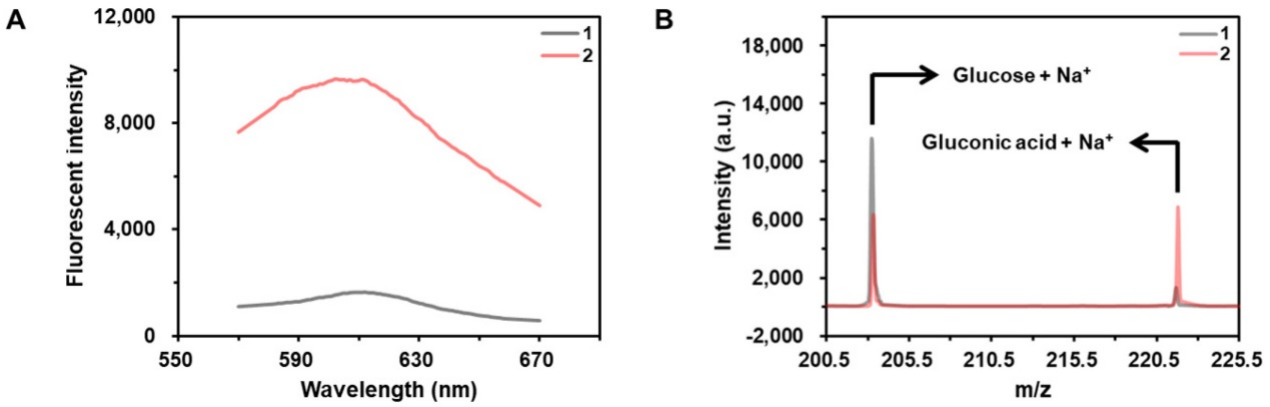
** Investigation of glucose oxidase-like activity of CeO_2_ NP.** (A) Fluorescent signals from the samples containing (1) hydroethidine and (2) hydroethidine and CeO_2_ NP. The concentrations of hydroethidine and CeO_2_ NP were 0.1 wt% and 1 µg/mL, respectively. (B) MALDI TOF spectra of the samples containing (a) glucose and (b) glucose and CeO_2_ NP. Sodium adduct peaks of glucose and gluconic acid appeared at m/z equivalent to *203* and *221*, respectively. The concentrations of glucose and CeO_2_ NP were *60* mM and *0.1* wt%, respectively.

As shown in the results of Figure 2A, the fluorescent signal from hydroethidine, an O_2_•^-^-specific fluorescent material, is highly enhanced in the presence of CeO_2_ NP under the acidic condition, demonstrating the production of O_2_•^-^. In addition, the results obtained by the matrix-assisted laser desorption/ionization time-of-flight (MALDI TOF) confirms that the glucose is converted to gluconic acid in the presence of CeO_2_ NP (Figure 2B).

We also investigated the kinetic parameters of CeO_2_ NPs by using the Michaelis-Menten model. As illustrated in Figure S1, Michaelis-Menten and Lineweaver-Burk plots were drawn by measuring the reaction velocities for the CeO_2_ NP-catalyzed glucose oxidation from the samples containing glucose at varying concentrations. The results show that the Michaelis-Menten constant (K_m_), maximum reaction velocity (V_max_), and catalytic constant (k_cat_) of CeO_2_ NP with 2 nm diameter were 5.93 mM, 0.59 μM/s, and 10.09 s^-1^, respectively, and these values are almost comparable to those of the glucose oxidase enzyme, demonstrating the high glucose oxidase-like activity of CeO_2_ NPs (Table 1) 34. Considering that the CeO_2_ NPs are inorganic nanomaterials with higher stability than the natural enzymes, we believe that CeO_2_ NP could be better suited for the practical applications. Furthermore, we also examined the effect of particle size, pH, and temperature on the glucose oxidase-like activity of CeO_2_ NP by measuring the glucose level difference defined as L_0_ - L where L_0_ and L are glucose levels before and after CeO_2_ NP-catalyzed glucose oxidation reaction, respectively. As shown in Figure S2A, the glucose oxidase-like activity increased with decreasing size of CeO_2_ NP, which is consistent with the previous report that the smaller CeO_2_ NP shows the enhanced catalytic activity 35-37. Moreover, the results in Figure S2B and S2C show that CeO_2_ NP exhibits the highest glucose oxidase-like activity under pH 4 and 20 °C.

### Detection feasibility

**Figure 3:**
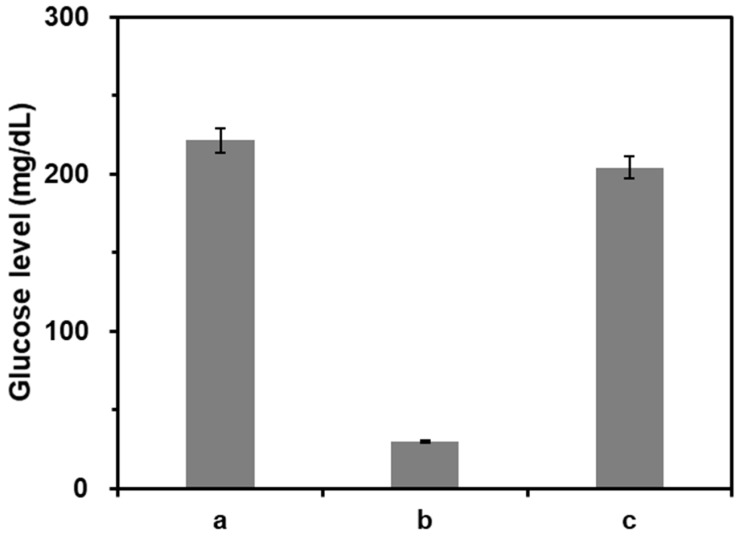
** Feasibility of PGM-based label-free read-out of PCR amplification.** The glucose levels were measured from reaction solutions containing (a) glucose only, (b) glucose and CeO_2_ NP, and (c) glucose, CeO_2_ NP, and purified DNA amplicon. The signal-to-noise ratios were calculated by dividing M by S where M and S are mean and standard deviation of the glucose levels based on triplicate measurements, which were (a) 28.5, (b) 41.7, and (c) 28.8. The concentrations of glucose, CeO_2_ NP, and purified DNA amplicon were *60* mM, *0.1* wt%, and *100* nM, respectively.

We first verified the feasibility of this strategy by measuring glucose levels from the samples at different reaction conditions. As shown in the results of Figure 3, the glucose level was significantly reduced by the presence of CeO_2_ NP due to its intrinsic glucose oxidase-like activity (b) compared to that in the absence of CeO_2_ NPs (a). On the other hand, when purified DNA amplicons was applied, the initial high glucose level was maintained (c). Notably, these results matched well with the Michaelis-Menten kinetic parameters. As shown in Table 1 and Figure S1, V_max_ decreased while K_m_ increased in the presence of purified DNA amplicon demonstrating the reduction of the efficiency for CeO_2_ NP-catalyzed glucose oxidation reaction, leading to the smaller catalytic constant (k_cat_) compared to that in the absence of DNA amplicon.

**Figure 4:**
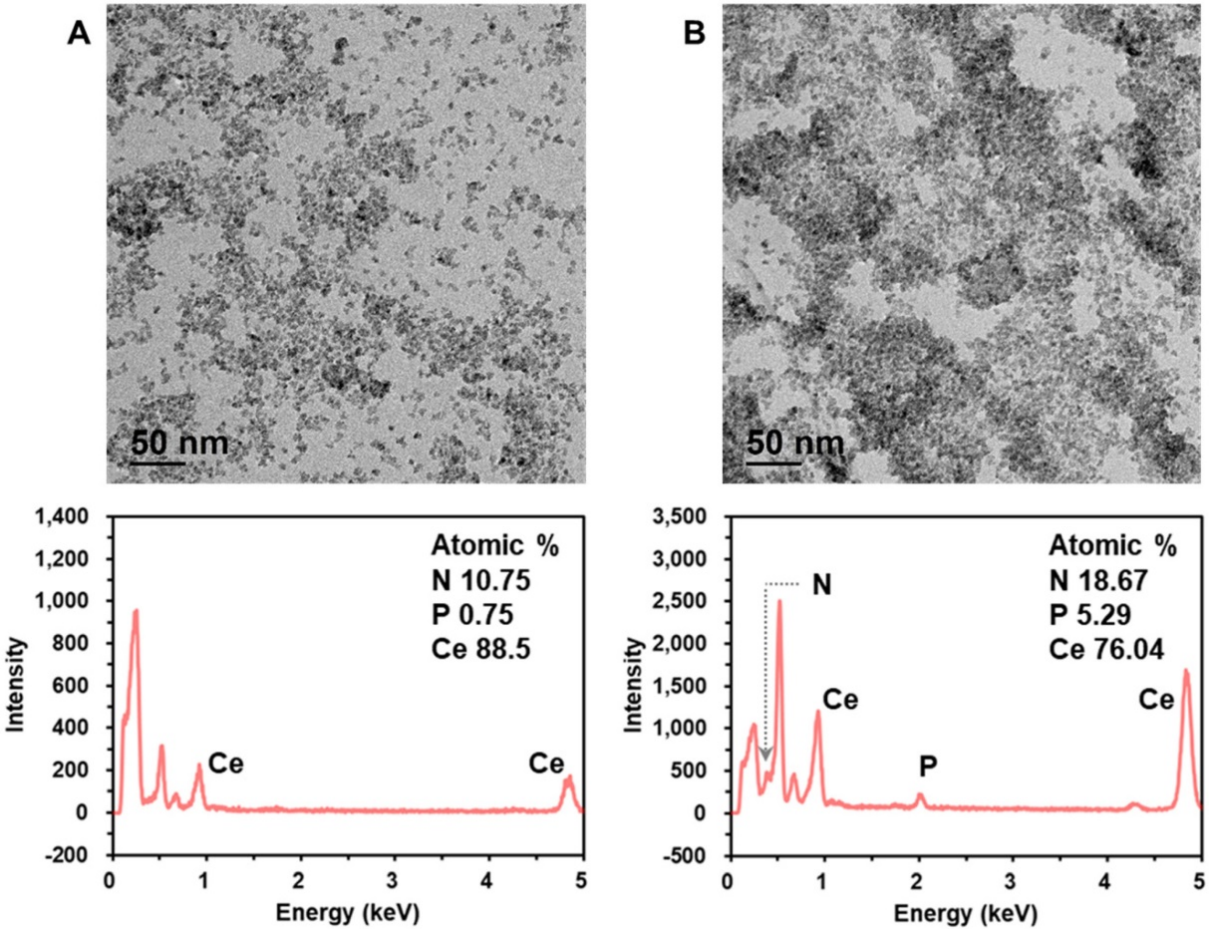
** Investigation of the mechanism for DNA amplicon binding-induced aggregation of CeO_2_ NP.** TEM images and corresponding EDS spectra of CeO_2_ NP in the (A) absence and (B) presence of purified DNA amplicon. The concentrations of CeO_2_ NP and purified DNA amplicon were *0.1* wt% and *100* nM, respectively.

Next, we characterized the crystalline nature and surface state of the employed CeO_2_ NP by analyzing X-ray diffraction (XRD) and X-ray photoelectron spectroscopy (XPS) pattern. The XRD and XPS spectrum shown in Figure S3 are in good agreement with those in the previous reports 30,33. In addition, we investigated the mechanism for DNA amplicon binding-induced aggregation of CeO_2_ NPs by using transmission electron microscopy (TEM), energy dispersive X-ray spectroscopy (EDS), zeta potential, dynamic light scattering (DLS), and ultraviolet-visible spectroscopy (UV-Vis) analyses. The TEM images and EDS spectra in Figure 4 clearly show that the CeO_2_ NPs were well dispersed in the absence of DNA amplicons, while the presence of purified DNA amplicons induced the aggregation of CeO_2_ NPs. In addition, the results of zeta potential (Figure S4A), DLS analysis (Figure S4B), EDS mapping (Figure S4C), and ultraviolet-visible spectroscopy (UV-Vis) analysis (Figure S5) supported our assumption that the surface of CeO_2_ NP shows positive charge which might results from the two oxidation states, Ce^3+^ and Ce^4+^, and thus the negatively charged DNA amplicons bind to the surface of CeO_2_ NPs, inducing the aggregation of CeO_2_ NPs 30-33.

### Quantitative analysis of DNA amplicons

The reaction conditions were then optimized by examining the change of glucose level defined as L/L_0_ where L_0_ and L are glucose levels from the reaction solutions in the absence and presence of purified DNA amplicons, respectively. The results in Figure S6 show that the optimal concentrations of CeO_2_ NP and glucose were 0.1 wt% and 60 mM, respectively. In addition, the optimal times for DNA amplicon incubation and glucose oxidation were 2 and 1.5 min, respectively, which were employed for further experiments (Figure S7).

Under the optimized conditions, the capability of this strategy to quantitatively determine purified DNA amplicons was investigated by measuring glucose levels from the reaction solutions containing purified DNA amplicons at varying concentrations (Figure S8). The results show that the glucose level increased as the concentration of purified DNA amplicon increased in the range from 5 to 100 nM with excellent linear relationship (R^2^ = 0.9983). Importantly, it should be noted that the typical concentration range of DNA amplicon produced by PCR is from 10 to 100 nM, which is fully covered by the dynamic range of the proposed method 19,38. In addition, it was confirmed that the glucose level increased as the concentration of the bound DNA amplicon increased, demonstrating the direct effect of the binding of DNA amplicon on the reduction of the efficiency for CeO_2_ NP-catalyzed glucose oxidation reaction.

### Sensitivity and selectivity for target DNA detection

**Figure 5:**
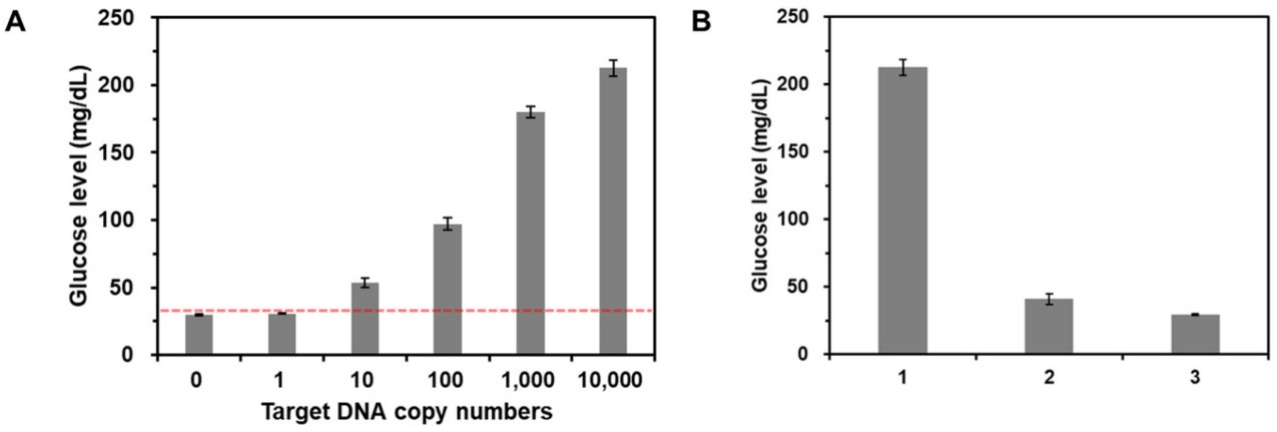
** Sensitivity and selectivity for target DNA detection.** (A) The glucose levels were measured from the reaction solutions containing target gDNA at varying amounts. The red dotted line is the threshold line defined as M + 3S where M and S are mean and standard deviation of glucose level obtained from the negative control without target DNA. The signal-to-noise ratios were calculated by dividing M by S where M and S are mean and standard deviation of the glucose levels based on triplicate measurements, which were 41.7, 53.1, 15.3, 21.2, 45, and 35.3 for the reaction solution with 0, 1, 10, 10^2^, 10^3^, and 10^4^ copies target gDNA, respectively. The concentrations of glucose and CeO_2_ NP were *60* mM and *0.1* wt%, respectively. (B) The glucose levels were measured from the reaction solutions containing (1) target gDNA (*E. coli*), (2) non-target gDNA (HBV), and (3) no target DNA. The signal-to-noise ratios were calculated by dividing M by S where M and S are mean and standard deviation of the glucose levels based on triplicate measurements, which were (1) 35.3, (2) 10.1, and (3) 41.7. The concentrations of glucose, CeO_2_ NP, and target or non-target gDNA were *60* mM, *0.1* wt%, and *200* copies/µL, respectively.

To examine the sensitivity of this method, target gDNA derived from *E. coli* with different copy numbers (0 - 10^4^ copies) were amplified by PCR, which was then purified and incubated with CeO_2_ NPs. As shown in Figure 5A, the glucose level increased as the copy number of target gDNA increased. Importantly, the glucose level obtained from the reaction solution containing *10* copies of target DNA was clearly distinguished from the threshold line (red dotted line) defined as M + 3S where M and S are mean and standard deviation of glucose level obtained from the negative control without target gDNA, demonstrating high sensitivity of this strategy compared to those of previous methods (Table S2). Furthermore, in the same manner, we also successfully determined the target gDNA in the human serum down to *500* copies (Figure S9).

Furthermore, we also investigated the selectivity of this strategy by measuring the glucose levels from the reaction solutions containing non-target gDNA derived from Hepatitis B virus (HBV). The results in Figure 5B show that the glucose level in the presence of target gDNA (*E. coli*) (1) is distinctly higher than those in the presence of non-target gDNA (HBV) and from the negative control without target gDNA (c), verifying the high selectivity for target DNA detection.

### Direct read-out of PCR amplification without post-purification steps

**Figure 6:**
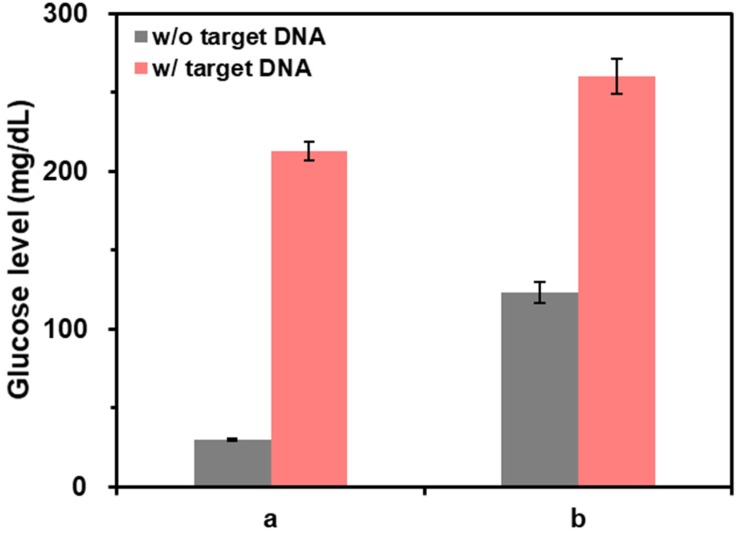
** The effect of post-purification step on the detection of target DNA.** The glucose levels were measured from samples containing (a) purified and (b) non-purified PCR solution. The signal-to-noise ratios were calculated by dividing M by S where M and S are mean and standard deviation of the glucose levels based on triplicate measurements, which were (a) 41.7 and 35.3 and (b) 18.1 and 23.5 for the reaction solution in the absence and presence of target DNA, respectively. The concentrations of glucose, CeO_2_ NP, and target DNA were *60* mM, *0.1* wt%, and 200 copies/µL, respectively.

Finally, the capability of this method for the direct read-out of PCR amplification without post-purification of PCR solution was examined by measuring the glucose levels from the purified and non-purified PCR solutions. As shown in the results of Figure 6, when the PCR solutions were purified (a), the glucose level in the presence of target DNA was clearly distinguished from the one in the absence of target DNA. In contrast, when PCR solutions were not purified (b), the glucose level in the absence of target DNA was not decreased as effectively as the one in the case of purified reaction solutions. This result was attributed to the presence of DNA primers, dNTPs, and DNA polymerase in PCR solutions that can induce the aggregation of CeO_2_ NPs reducing their catalytic activity. However, the difference of glucose level in the absence and presence of target DNA was still high enough to determine the target DNA, demonstrating the capability of this method to directly examine PCR results without post-purification steps.

## Conclusions

In this study, we developed a PGM-based label-free method for the on-site read-out of PCR amplification using glucose oxidase-like activity of CeO_2_ NPs that correlates the glucose level with the amount of DNA amplicon. With the proposed strategy, the target DNA was sensitively detected down to *10* copies with the high selectivity. In addition, after the completion of PCR, the result was rapidly examined in less than *5* min without any tedious and labor-intensive procedures. Importantly, this strategy employs the PGM as a detection component which would enable its facile application in the POC settings. It is noteworthy that this is the first report to discover the glucose oxidase-like activity of CeO_2_ NPs and utilize their activity for the label-free analysis of PCR amplification. Based on the advantageous features of rapidity, simplicity, cost-effectiveness, and portability, we expect that the devised system could serve as a core platform for the on-site read-out of PCR result.

## Supplementary Material

Supplementary figures and tables.

## Figures and Tables

**Table 1:** Comparison of Michaelis-Menten kinetic parameters of (a) CeO_2_ NP, (b) CeO_2_ NP/DNA amplicon complex, and (c) glucose oxidase. The concentrations of CeO_2_ NP, purified DNA amplicon, and glucose oxidase were 70, 100, and 70 nM

Material	K_m_ (mM)	V_max_ (μM/s)	k_cat_ (s^-1^)
a	5.93	0.59	10.09
b	11.67	0.33	5.69
c	4.87	0.69	9.71

## Abbreviations

CeO_2_ NP: cerium oxide nanoparticle
DHB: 2,5-dihydroxybenzoic acid
DLS: dynamic light scattering
dNTP: deoxynucleoside triphosphate
EDS: energy-dispersive X-ray spectroscopy
gDNA: genomic DNA
MALDI TOF: matrix-assisted laser desorption/ionization time-of-flight
PCR: polymerase chain reaction
PGM: personal glucose meter
POC: point-of-care
TEM: transmission electron microscopy
UV-Vis: ultraviolet-visible spectroscopy
XRD: X-ray diffraction
