# Supplementary material for: Glucose oxidase-like activity of cerium oxide nanoparticles: use for personal glucose meter-based label-free target DNA detection
Source: Theranostics. 2020 Mar 15;10(10):4507–14. doi: 10.7150/thno.41484 (PMC7150472; doi:10.7150/thno.41484)
Supplement: Supplementary file 1 — Supplementary figures and tables. [file thnov10p4507s1.pdf]

Supplementary Material for

Glucose oxidase-like activity of cerium oxide nanoparticles: use for personal glucose meter-based label-free target DNA detection

Hyo Yong Kim[a], Ki Soo Park[b,*], and Hyun Gyu Park[a,*]

[a] Department of Chemical and Biomolecular Engineering (BK21+ Program), KAIST, 291 Daehak-ro, Yuseong-gu, Daejeon 34141, Republic of Korea.

[b] Department of Biological Engineering, College of Engineering, Konkuk University, Seoul 05029, Republic of Korea

[*] To whom correspondence should be addressed.

E-mail: hgpark@kaist.ac.kr (H.G. Park); Phone: +82-42-350-3932; Fax: +82-42-350-3910.

E-mail: kskonkuk@gmail.com (K.S. Park); Phone: +82-2-350-3742; Fax: +82-2-350-3742.

Table S1. Oligonucleotide sequences employed in this study.

Name	Sequence (5' → 3')
Forward primer	AGA GTT TGA TCC TGG CTC AG
Reverse primer	TTA CCG CGG CTG CTG GC
DNA amplicon for *E. coli* 16S rRNA gene	AGA GTT TGA TCC TGG CTC AGA TTG AAC GCT GGC GGC AGG CCT AAC ACA TGC AAG TCG AAC GGT AAC AGG AAA CAG CTT GCT GTT TCG CTG ACG AGT GGC GGA CGG GTG AGT AAT GTC TGG GAA ACT GCC TGA TGG AGG GGG ATA ACT ACT GGA AAC GGT AGC TAA TAC CGC ATA ACG TCG CAA GAC CAA AGA GGG GGA CCT TCG GGC CTC TTG CCA TAG ATG TGC CCA GAT GGG ATT AGC TAG TAG GTG GGG TAA CGG CTC ACC TAG GCG ACG ATC CCT AGC TGG TCT GAG AGG ATG ACC AGC CAC ACT GGA ACT GAG ACA CGG TCC AGA CTC CTA CGG GAG GCA GCA GTG GGG AAT ATT GCA CAA TGG GCG CAA GCC TGA TGC AGC CAT GCC GCG TGT ATG AAG AAG GCC TTC GGG TTG TAA AGT ACT TTC AGC GGG GAG GAA GGG AGT AAA GTT AAT ACC TTT GCT CAT TGA CTT ACC CGC AGA AGA AGC ACC GGC TAA CTC CGT GCC AGC AGC CGC GGT AA

Table S2. Comparison of this strategy with previous PCR-based target DNA detection methods.

Material/Method	Signal	Detection limit (copy number)	Assay time (min)	Reference
Redox material-labeled DNA hairpin probe	Electrochemistry	10^2	*45*	[1]
Thiol-labeled primer and gold nanoparticle	Absorbance	10^2	*60*	[2]
Intercalating redox material and magnetic particle	Electrochemistry	10^6	*300*	[3]
DNA nanostructure	Electrochemistry	10^3	*160*	[4]
Glucose oxidase-like activity of CeO_2 NP	PGM	*10*	~ *5*	This work

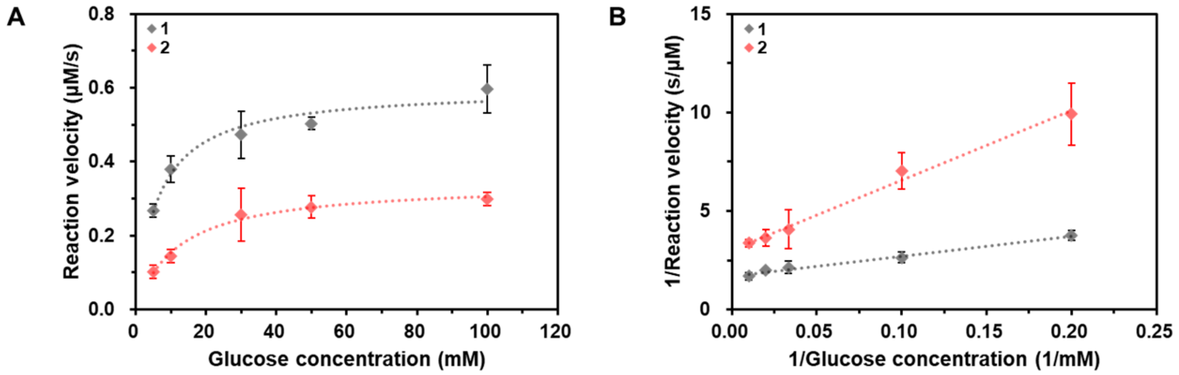

Figure S1. (A) Michaelis-Menten and (B) Lineweaver-Burk plots of CeO_2 NP-catalyzed glucose oxidation reaction in the (1) absence and (2) presence of purified DNA amplicon. The reaction velocity was calculated by dividing (L_0 - L) by the reaction time (*30* min), where L_0 and L are glucose levels before and after CeO_2 NP-catalyzed glucose oxidation reaction, respectively. The concentrations of CeO_2 NP and purified DNA amplicon were *70* and *100* nM, respectively.

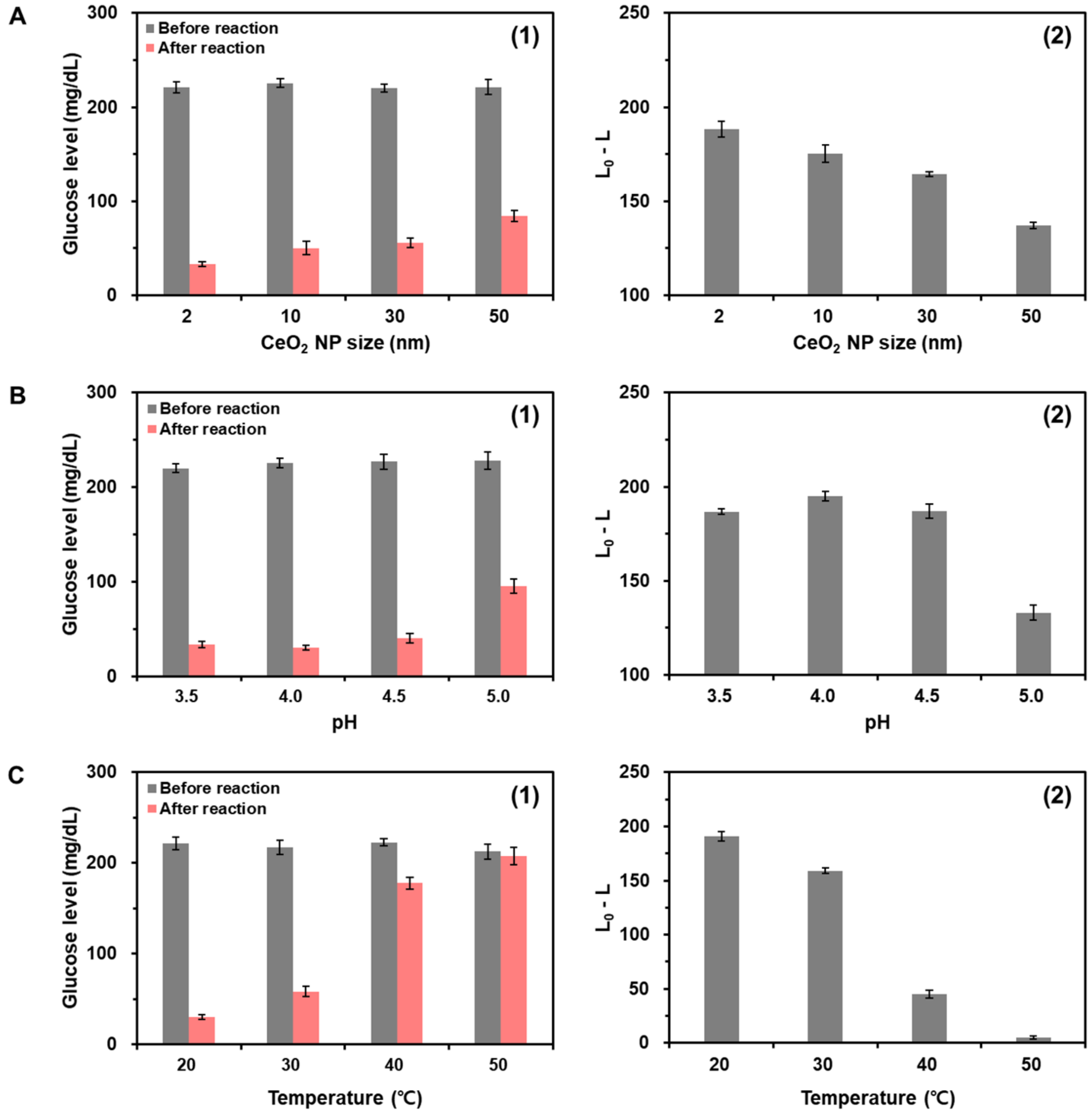

Figure S2. The effect of (A) particle size, (B) pH, and (C) temperature on the glucose oxidase-like activity of CeO_2 NP by (1) measuring glucose level and (2) calculating the glucose level difference defined as L_0 - L where L_0 and L are glucose levels before and after CeO_2 NP-catalyzed glucose oxidation reaction, respectively. The concentrations of CeO_2 NP and glucose were *0.1* wt% and *60* mM, respectively.

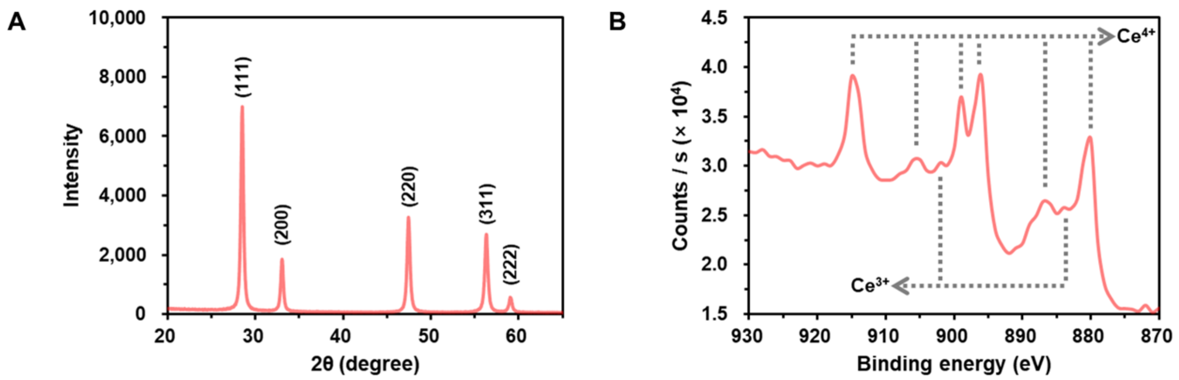

Figure S3. Characterization of CeO_2 NP by analyzing (A) XRD and (B) XPS spectrum.

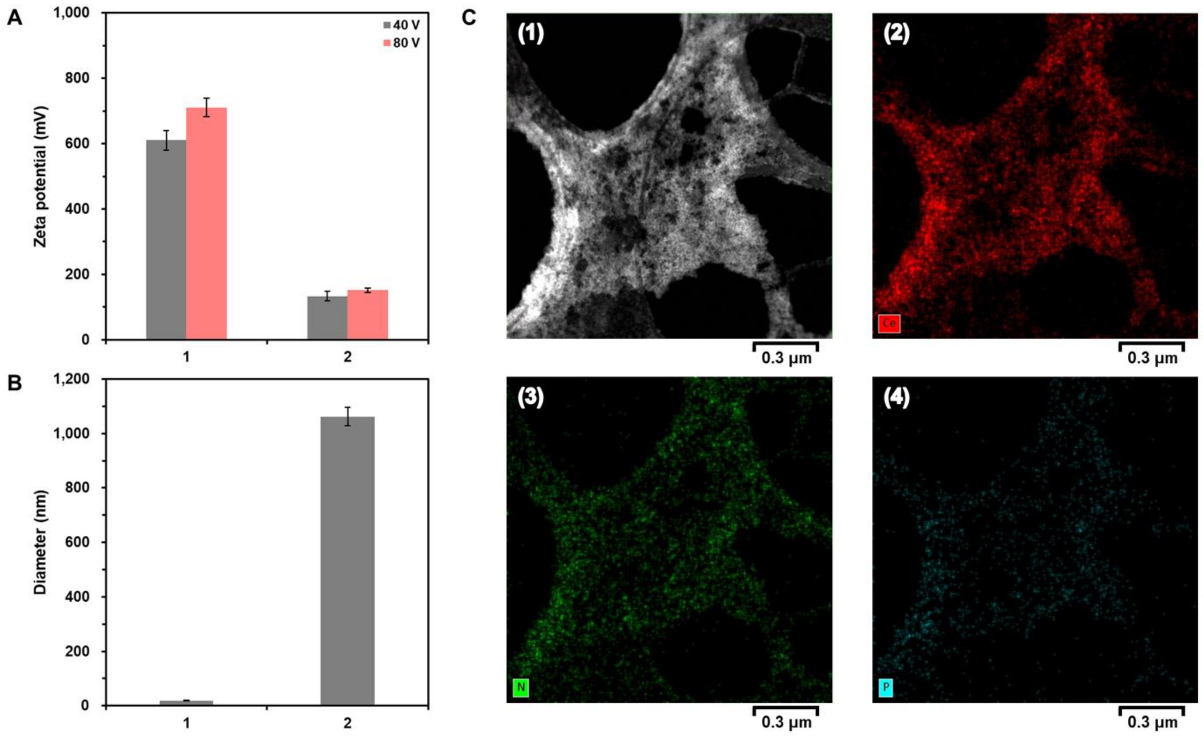

Figure S4. (A) Zeta potentials under different voltages (40 and 80 V) and (B) diameter of CeO_2 NP in the (1) absence and (2) presence of purified DNA amplicon. The concentrations of CeO_2 NP and purified DNA amplicon were *0.1* wt% and *100* nM, respectively. (C) EDS mapping for the confirmation of the binding of DNA amplicon onto CeO_2 NP. (1) TEM image of CeO_2 NP in the presence of purified DNA amplicon and corresponding EDS images of (2) cerium, (3) nitrogen, and (4) phosphorus element. The concentrations of CeO_2 NP and purified DNA amplicon were *0.1* wt% and *200* nM, respectively.

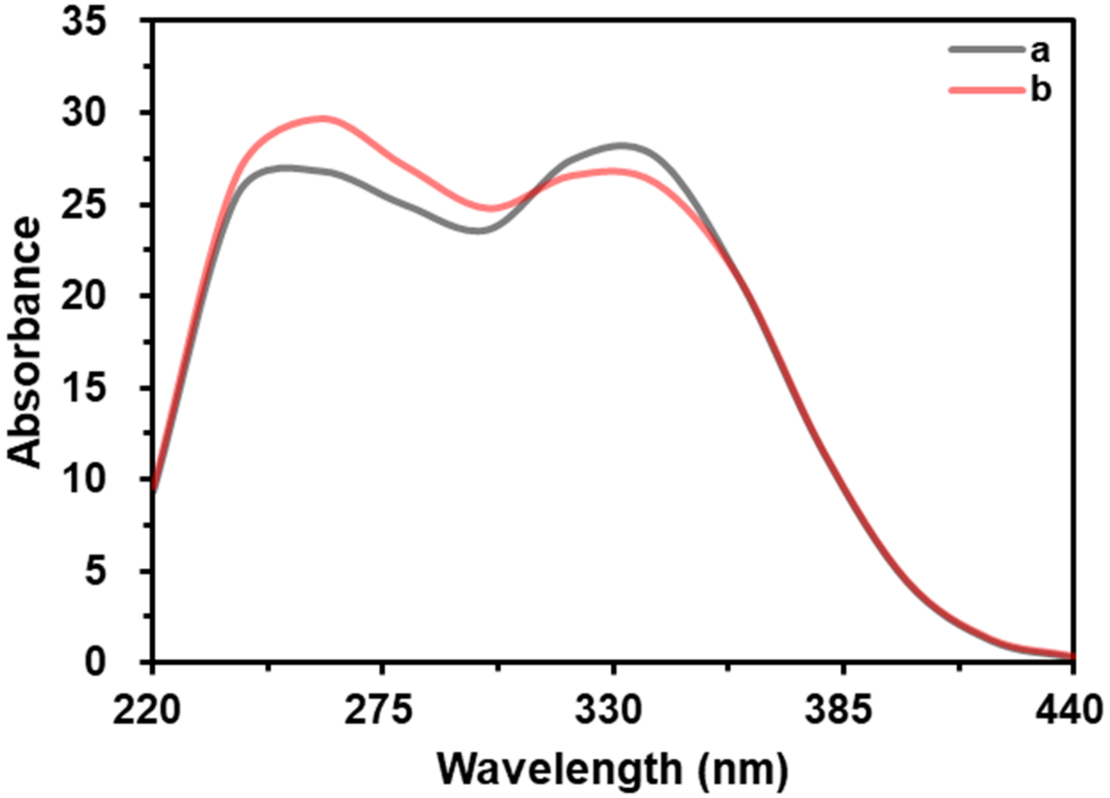

Figure S5. UV-Vis absorbance spectra of CeO_2 NP in the (a) absence and (b) presence of purified DNA amplicon. The absorbance peaks at *250* and *340* nm were from O^{2-} (2p) and Ce^{4+} (4f) orbitals in CeO_2 NP, respectively, and the one at *260* nm was from DNA. The concentrations of CeO_2 NP and purified DNA amplicon were *0.1* wt% and *100* nM, respectively.

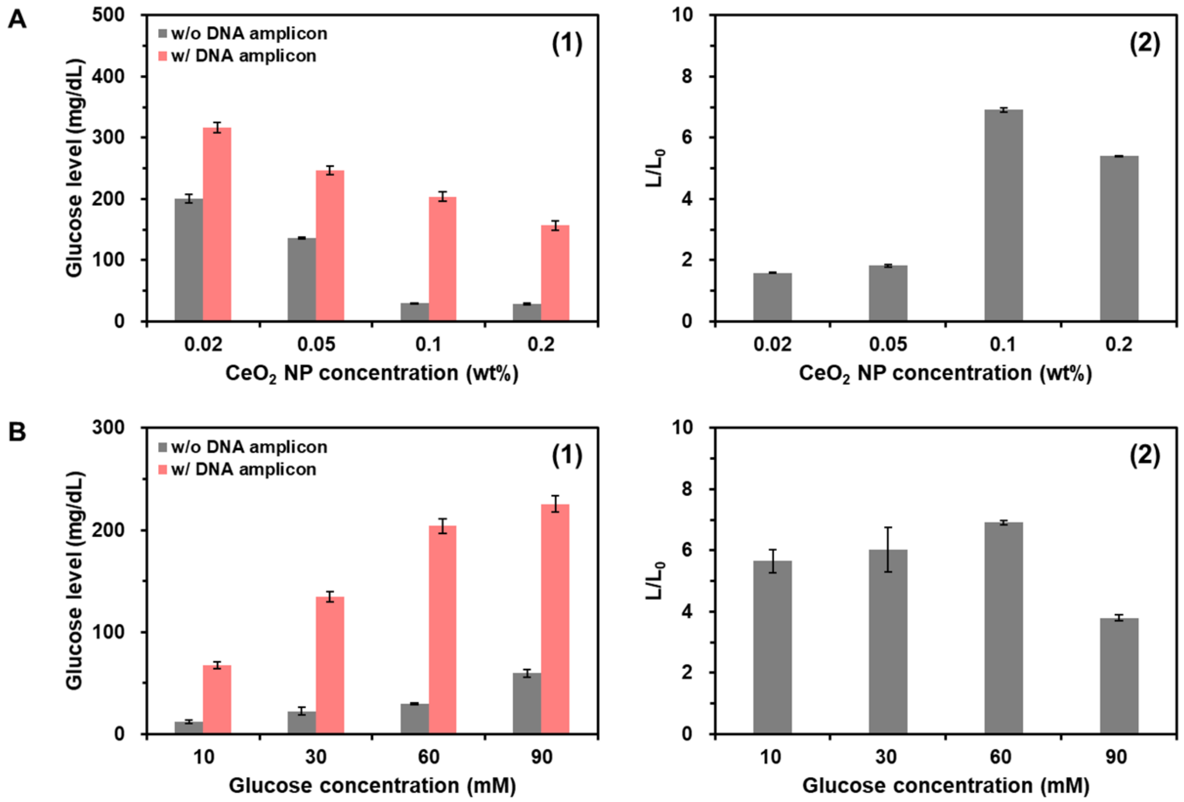

Figure S6. Optimization of (A) CeO_2 NP and (B) glucose concentration by (1) measuring glucose level and (2) calculating the change of glucose level defined as L/L_0 where L_0 and L are glucose levels from the reaction solutions in the absence and presence of purified DNA amplicon, respectively. The concentration of glucose for the optimization of (A) CeO_2 NP concentration was *60* mM. The concentration of CeO_2 NP for the optimization of (B) glucose concentration was *0.1* wt%. The concentration of purified DNA amplicon was *100* nM.

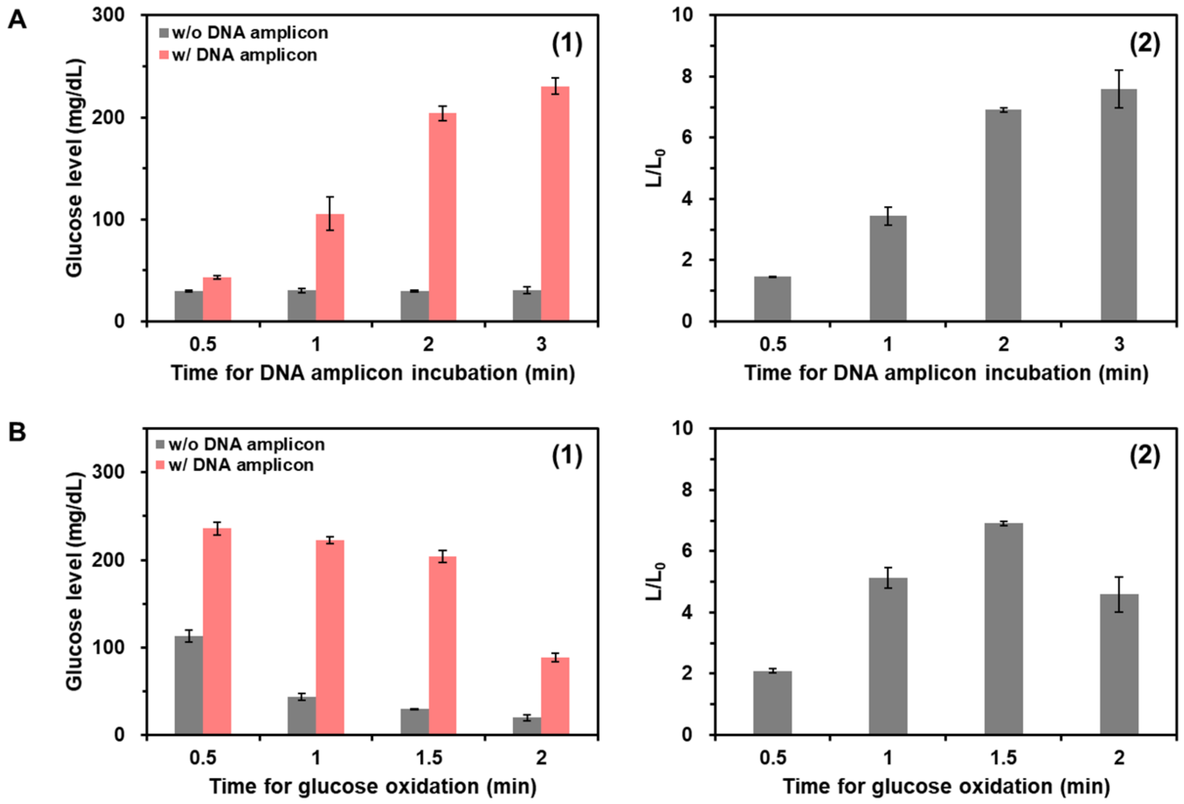

Figure S7. Optimization of the time for (A) DNA amplicon incubation and (B) glucose oxidation by (1) measuring glucose level and (2) calculating the change of glucose level defined as L/L_0 where L_0 and L are glucose levels from the reaction solutions in the absence and presence of purified DNA amplicon, respectively. The concentrations of glucose, CeO_2 NP, and purified DNA amplicon were *60* mM, *0.1* wt%, and *100* nM, respectively.

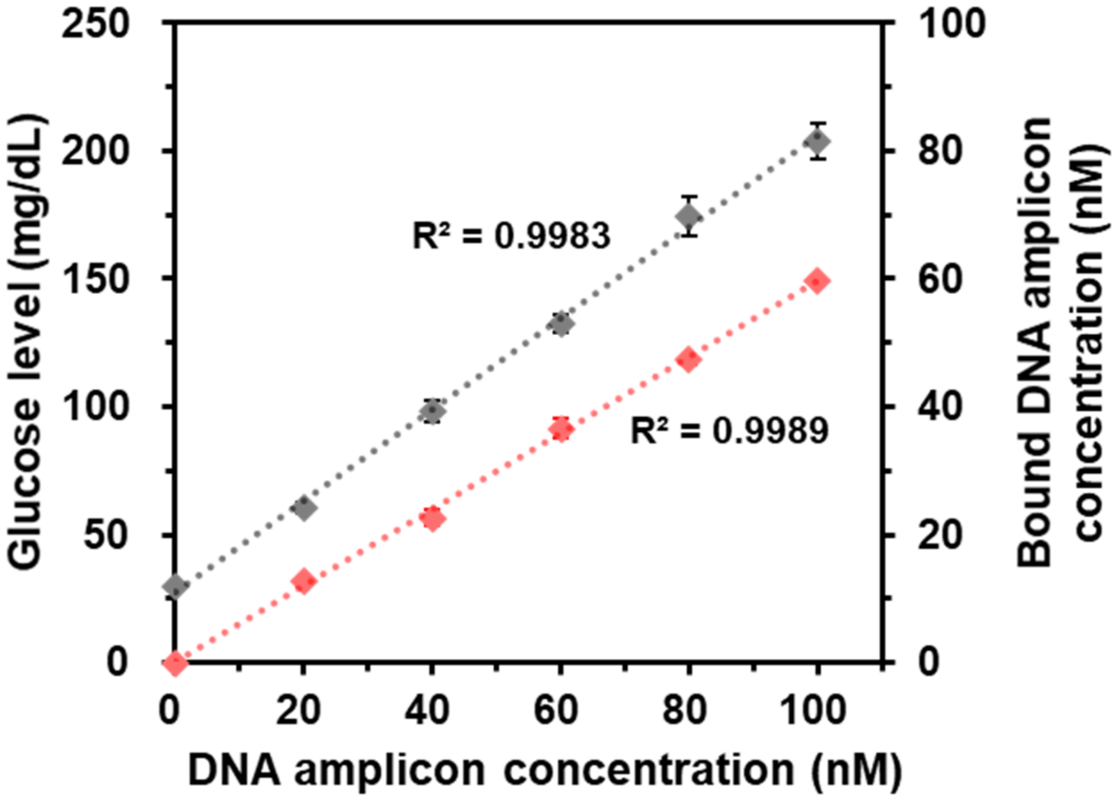

Figure S8. Quantitative analysis of DNA amplicons by measuring glucose levels (black line) and the concentration of the bound DNA amplicon (red line) from the reaction solutions containing purified DNA amplicons at varying concentrations. Bound DNA amplicon concentration was calculated by subtracting the concentration of DNA amplicon remained in the solution after the precipitation of CeO_2 NP/DNA amplicon complex at *10,000* g for *5* min from the initially added one. The concentrations of glucose and CeO_2 NP were *60* mM and *0.1* wt%, respectively.

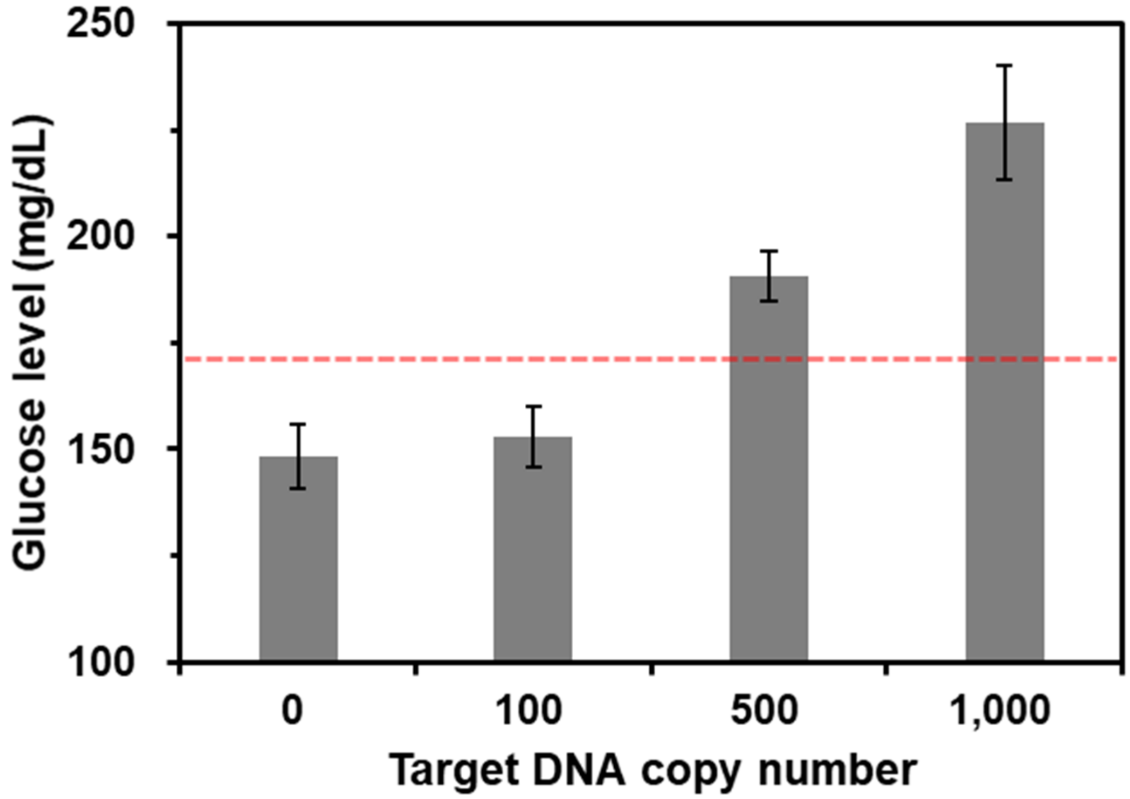

Figure S9. Determination of target DNA in the human serum (1%) by measuring the glucose levels from the reaction solutions containing target gDNA at varying amounts. The concentrations of glucose and CeO_2 NP were *60* mM and *0.1* wt%, respectively.

References

1. Lai RY, Lagally ET, Lee SH, Soh HT, Plaxco KW, Heeger AJ. Rapid, sequence-specific detection of unpurified PCR amplicons via a reusable, electrochemical sensor. Proc Natl Acad Sci U S A. 2006; 103: 4017-21.
2. Jung YL, Jung C, Parab H, Li T, Park HG. Direct colorimetric diagnosis of pathogen infections by utilizing thiol-labeled PCR primers and unmodified gold nanoparticles. Biosens Bioelectron. 2010; 25: 1941-6.
3. Luo X, Xu J, Barford J, Hsing IM. Magnetic particle based electrochemical sensing platform for PCR amplicon detection. Electrochem Commun. 2010; 12: 531-4.
4. Wen Y, Wang L, Xu L, Li L, Ren S, Cao C, et al. Electrochemical detection of PCR amplicons of Escherichia coli genome based on DNA nanostructural probes and polyHRP enzyme. Analyst. 2016; 141: 5304-10.
